# Healing the Ischaemic Heart: A Critical Review of Stem Cell Therapies

**DOI:** 10.31083/j.rcm2404122

**Published:** 2023-04-19

**Authors:** Devin Tonkin, Anthony Yee-Goh, Rajesh Katare

**Affiliations:** ^1^Department of Physiology, HeartOtago, School of Biomedical Sciences, University of Otago, 9010 Dunedin, New Zealand

**Keywords:** ischaemic heart disease, stem cells, clinical trials, pluripotent stem cells, adult stem cells, paracrine mechanisms

## Abstract

Ischaemic heart disease (IHD) remains the leading cause of mortality worldwide. Current pharmaceutical treatments focus on delaying, rather than preventing disease progression. The only curative treatment available is orthotopic heart transplantation, which is greatly limited by a lack of available donors and the possibility for immune rejection. As a result, novel therapies are consistently being sought to improve the quality and duration of life of those suffering from IHD. Stem cell therapies have garnered attention globally owing to their potential to replace lost cardiac cells, regenerate the ischaemic myocardium and to release protective paracrine factors. Despite recent advances in regenerative cardiology, one of the biggest challenges in the clinical translation of cell-based therapies is determining the most efficacious cell type for repair. Multiple cell types have been investigated in clinical trials; with inconsistent methodologies and isolation protocols making it difficult to draw strong conclusions. This review provides an overview of IHD focusing on pathogenesis and complications, followed by a summary of different stem cells which have been trialled for use in the treatment of IHD, and ends by exploring the known mechanisms by which stem cells mediate their beneficial effects on ischaemic myocardium.

## 1. Introduction

Despite global research efforts, cardiovascular disease (CVD) remains the leading cause of mortality worldwide [1]. The term CVD encompasses a multitude of cardiac pathologies including valvular defects, arrhythmias, vasculopathies and congenital malformations. Ischaemic heart disease (IHD) is the most prevalent form of CVD, characterised by insufficient blood supply to myocardium relative to oxygen demand [2]. This ischaemic imbalance results from the formation of atherosclerotic lesions in the tunica intima of coronary arteries, causing progressive stenosis of the coronary lumen and a corresponding decrease in blood flow [3].

As IHD progresses, functional cardiomyocytes are lost owing to ischaemia with remaining myocytes forced to hypertrophy to compensate and maintain heart function [4]. This progressive loss of cardiomyocytes often eventuates in chronic heart failure (CHF)—where the cardiovascular system is unable to supply blood to tissues at normal perfusion pressures. Furthermore, increasing coronary artery stenosis places the patient at risk of plaque rupture and acute coronary syndromes including myocardial infarction (MI), unstable angina and cerebrovascular accident [5]. Current pharmaceutical treatments only delay the progression of IHD, but are unable to reverse existing damage to the myocardium. The replacement of lost cardiovascular cells, along with improving the function and survival of remaining cells is vital for long-term improvement of cardiac function in patients with IHD. Presently, orthotopic heart transplantation is the only way to achieve this goal. However, a limited number of suitable donors and the need for lifelong immune system modulation limit the availability of allotransplantation [6].

Stem cells have garnered global attention as a therapy for a number of pathological conditions following their discovery in 1961 [7]. They have the theoretical potential to both halt the progression of IHD and reverse existing damage by replacing lost cardiac cells, improving the function of resident cells, and the release of beneficial paracrine factors. However, recent studies demonstrate that different stem cell types have varying efficacy in repairing the ischaemic heart [8, 9]. This review will first provide an overview of IHD focusing on pathogenesis and complications, followed by a summary of different stem cell populations trialled for the treatment of IHD and end by exploring the known mechanisms by which these stem cells mediate their beneficial effect on the ischaemic myocardium.

## 2. Pathogenesis of IHD & Progression to CHF

**Fig. 1.**
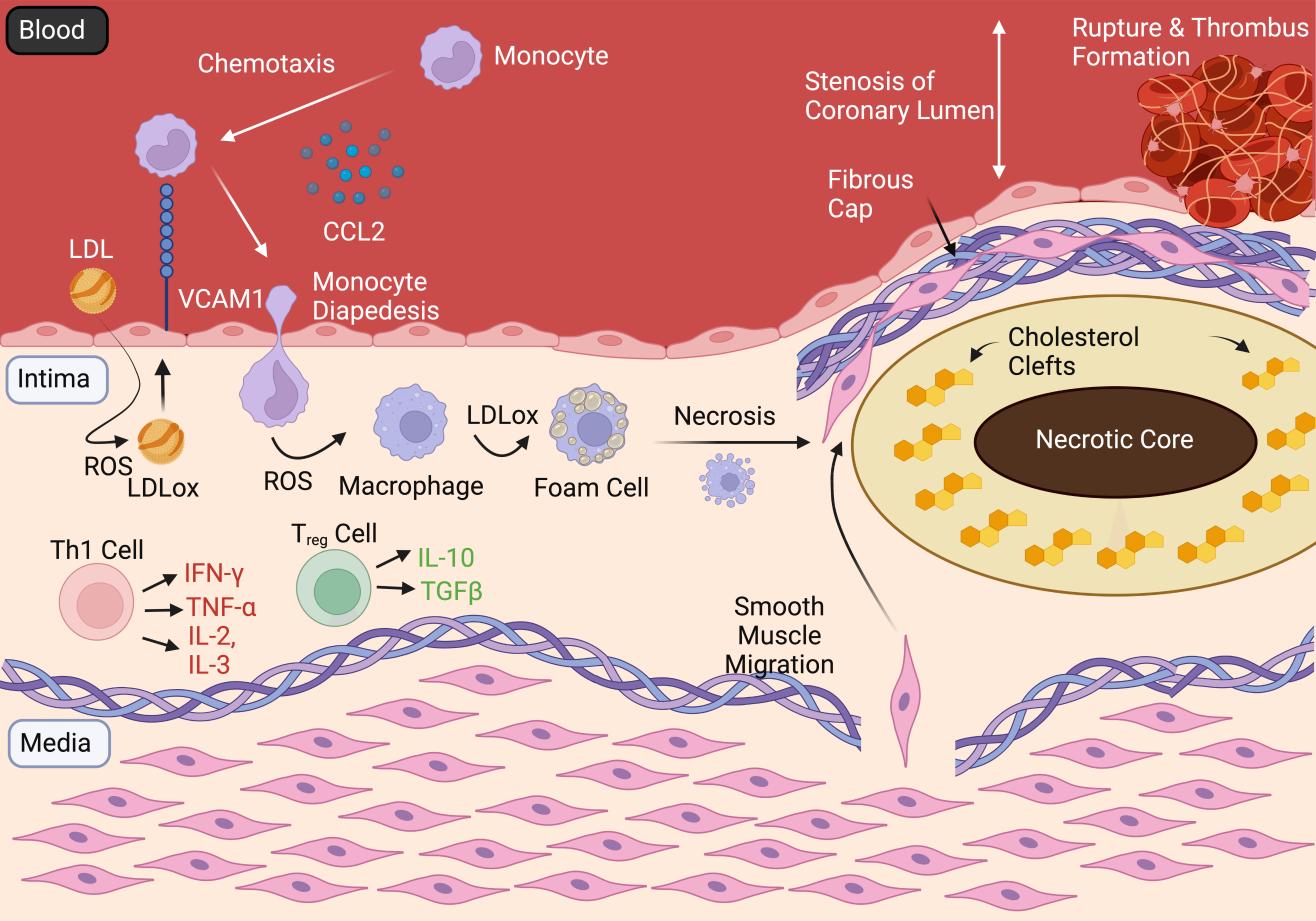
**Pathogenesis and Complications of IHD**. Figure summarising the development and progression of the atherosclerotic plaque, which obstructs the coronary lumen and if unstable—can rupture, with subsueqent thrombus formation occluding the affected coronary artery—resulting in acute coronary syndromes. LDL, low density lipoprotein; VCAM1, vascular cell adhesion molecule 1; CCL2, chemoattractant protein 1; ROS, reactive oxygen species; LDLox, oxidised LDL; IFN-Y, interferon gamma; TNF-α, tumour necrosis factor alpha; IL-2, interleukin 2; IL-3, interleukin 3; IL-10, interleukin 10; TGFβ, transforming growth factor beta; Th1, T helper type 1; $T_{\text{reg}}$, regulatory T cell.

IHD is driven by the formation of atherosclerotic plaques within the tunica intima of coronary arteries, occluding the coronary lumen (Fig. 1). This atherogenesis is thought to stem from a combination of endothelial dysfunction and hypercholesterolaemia [10, 11]. Risk factors for these states include diabetes mellitus, hypertension, smoking, obesity and a lipid-rich diet [12]. Together, these states act to increase the vascular permeability of coronary arteries, allowing the migration of lipids including the cholesterol rich low-density lipoprotein (LDL) through the endothelial lining, into the tunica intima [13]. Here, LDL particles are oxidised by reactive oxygen species (ROS) and become pro-inflammatory, driving the activation of endothelium and expression of vascular adhesion molecule-1 (VCAM-1) and intercellular adhesion molecule-1 (ICAM-1) on the endothelial cell surface [14, 15]. Chemotactic agents including chemokine ligand 2 (CCL2) are released from activated endothelium and recruit mast cells, neutrophils and monocytes which bind to the aforementioned endothelial cell surface adhesion molecules [16].

Following attachment to adhesion molecules, monocytes migrate into the tunica intima, where they differentiate into macrophages in the presence of ROS. These macrophages are involved in further leucocyte recruitment and cytokine release in addition to phagocytosis of oxidised LDL, after which they are referred to as foam cells [17]. Accumulation of foam cells forms a ‘fatty streak’ - the earliest gross pathological sign of atherosclerosis. Over time these foam cells necrose through apoptosis, creating the characteristic ‘necrotic core’ of the atherosclerotic plaque [18]. New blood vessels primarily from the tunica adventitia can grow into the base of atherosclerotic lesions. This can further advance plaque growth as these vessels provide yet another avenue for monocytes and other immune cells to reach the plaque [19]. Meanwhile, adjacent endothelial and smooth muscle cells (SMCs) secrete cytokines and growth factors, causing SMCs to migrate to the luminal side of the vessel wall. This leads to the formation of a fibrous cap composed of collagen, SMCs, macrophages and T lymphocytes (Fig. 1) [20].

Depending on the progression and stability of the atherosclerotic plaque, IHD can progress to develop further complications. The degree of plaque stability is directly proportional to the thickness of the fibrous cap surrounding the necrotic, lipid-rich core [21]. Stable plaques result in stable angina, characterised by ischaemic chest pain induced by exertional stress. In the absence of myocardial scarring, stable angina is rarely fatal and is usually relievable with rest or nitroglycerin [22, 23].

Acute coronary syndromes (ACS) result from atherosclerotic plaque rupture, with subsequent thrombus formation leading to incomplete or total occlusion of the coronary lumen [24, 25]. ACS are medical emergencies, drastically reducing blood flow to the myocardium, resulting in unstable angina and MI. Myocardial infarction can be further classified into ST-elevation myocardial infarction (STEMI) or Non ST-elevation myocardial infarction (NSTEMI). Both NSTEMI and STEMI are characterised by myocardial necrosis subsequent to the onset of sudden ischaemia [26].

**Fig. 2.**
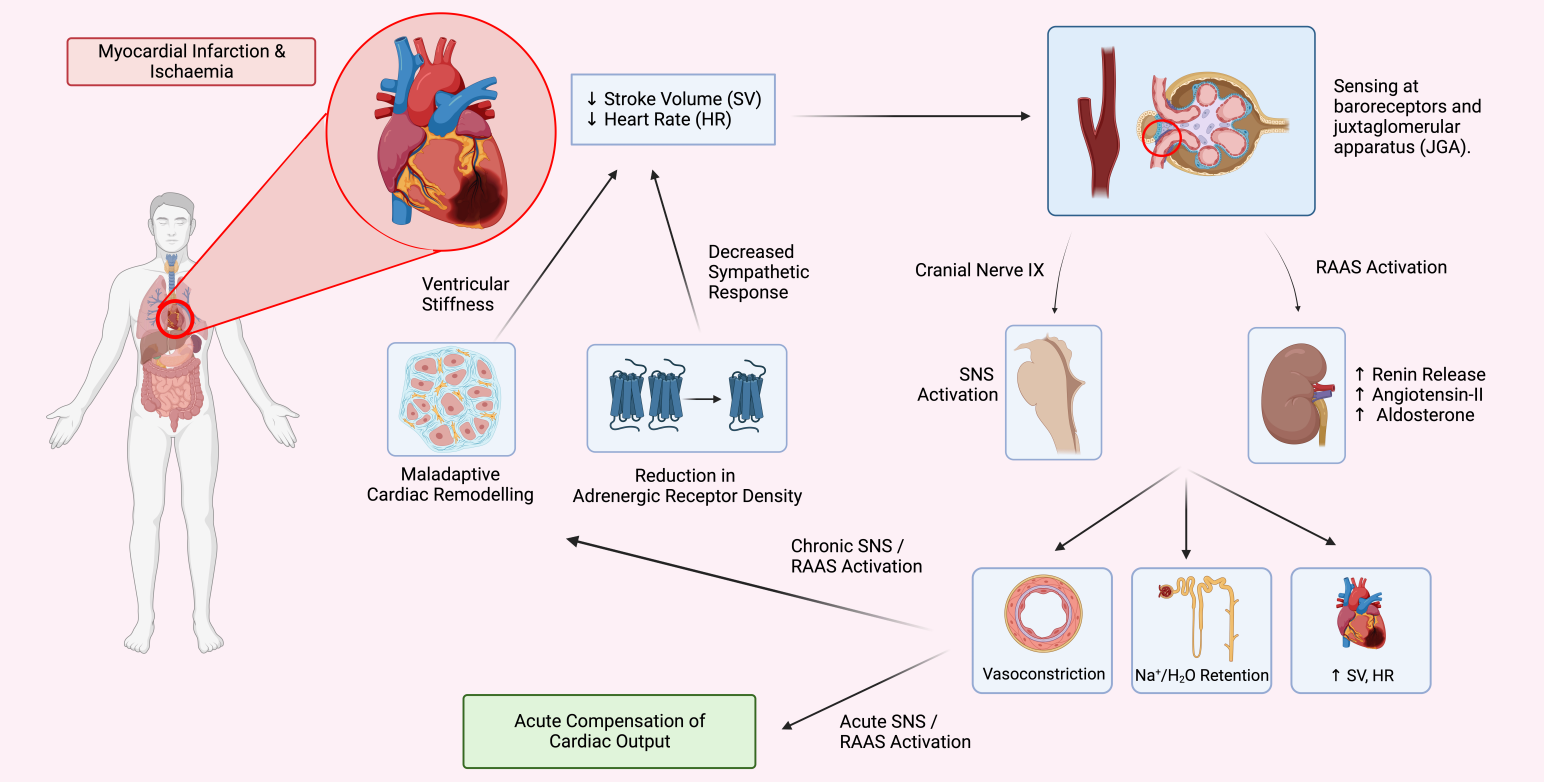
**The Cycle of Heart Failure**. In response to myocardial injury, neurohumoral mechanisms are activated, compensating for decreased cardiac output. However, in the long term, these mechanisms cause further wall stress and cardiac remodelling, further damaging cardiac tissue and advancing the progression of heart failure. RAAS, renin-angiotensin-aldosterone system; SNS, sympathetic nervous system.

In the event of MI, both the renin-angiotensin-aldosterone system (RAAS) and the sympathetic nervous system (SNS) become activated to maintain adequate perfusion of the vital organs [27, 28] (Fig. 2). This is achieved by inducing vasoconstriction of systemic arterioles, increasing total peripheral resistance (TPR), and increasing renal reabsorption of sodium and water to elevate systemic blood pressure. Angiotensin II, the key component of the RAAS system increases the production of ROS within the heart through NADPH oxidase 2 (NOX2) activation which in turn activates downstream pathways involved in cardiac hypertrophy including protein kinase B (Akt), nuclear factor kappa B (NF-kB) and extracellular signal-related kinase (ERK1/2) signalling [29].

Further, an inflammatory response simultaneously occurs by initiating the recruitment of macrophages to the infarcted area to remove dead cells and matrix debris by phagocytosis [30]. Following MI, resident cardiac fibroblasts (CFs) become pro-inflammatory, increasing their secretion of pro-inflammatory cytokines including interleukin-1 (IL-1). These CFs undergo differentiation into myofibroblasts, increasing the rate of extracellular matrix (ECM) deposition and scar formation [31, 32].

Activation of these compensatory mechanisms are crucial to sustain cardiac output during the initial phase following MI. However, sustained activation of these pathways can eventuate in heart failure. Sustained increases in RAAS and SNS activation increase ventricular wall stress, contributing to maladaptive cardiac remodelling, reducing ventricular function (Fig. 2) [33, 34, 35].

Furthermore, activation of the SNS desensitises β_1_ adrenergic receptors which in turn reduces cardiac force in response to a given sympathetic input. As a consequence, SNS activity can further increase, desensitising more receptors and reducing their density [36]. Although the deposition of fibrotic tissue maintains the integrity and basic structure of the left ventricular chamber following MI, it compromises cardiac contractility in the long-term through an increase in cardiac stiffness and a corresponding reduction in tissue elasticity [37].

## 3. Current Treatments for IHD

The management of IHD necessitates a combination of lifestyle and pharmacological approaches, with the ongoing involvement of a multidisciplinary healthcare team. The primary aims of IHD management are symptom relief and prevention of disease progression. Lifestyle interventions aim to control for modifiable risk factors including a sedentary lifestyle, hyperlipidaemia, smoking and hypertension [12]. These factors may be controlled by regular exercise, smoking cessation, weight loss and the maintenance of a healthy diet. A recent systematic review of structured lifestyle modification programmes highlighted significant reductions in all-cause mortality, cardiac mortality and cardiac re-admissions, emphasising the importance of lifestyle modifications [38]. However, these changes may prove challenging to elicit in practice due to several barriers the patient may be facing.

Pharmaceutical interventions for IHD range from anti-hypertensives including angiotensin converting enzyme (ACE) inhibitors, antidyslipidaemics such as atorvastatin along with anticoagulant therapy to lower the risk of thrombus formation [39]. In the case of acute MI, the immediate aims of treatment are to promote reperfusion to the myocardium, reducing infarct size. This can be achieved with prompt fibrinolytic, antiplatelet and antithrombotic agents, along with percutaneous coronary intervention (PCI) to restore blood flow mechanically via stenting the affected coronary artery [40].

If the patient has progressed to CHF, the goal of pharmacological therapy is to improve cardiac contractility and to reduce fluid overload [41]. Orthotropic heart transplantation remains the only curative treatment, unfortunately limited by low donor availability [42]. None of the current treatments are able to reverse the disease process or restore lost cardiac cells. As a result, interest has grown globally in seeking novel therapies for IHD, among which cell-based therapies have garnered attention due to their potential to promote cardiac repair and regeneration.

## 4. Stem Cell Therapy for IHD

**Fig. 3.**
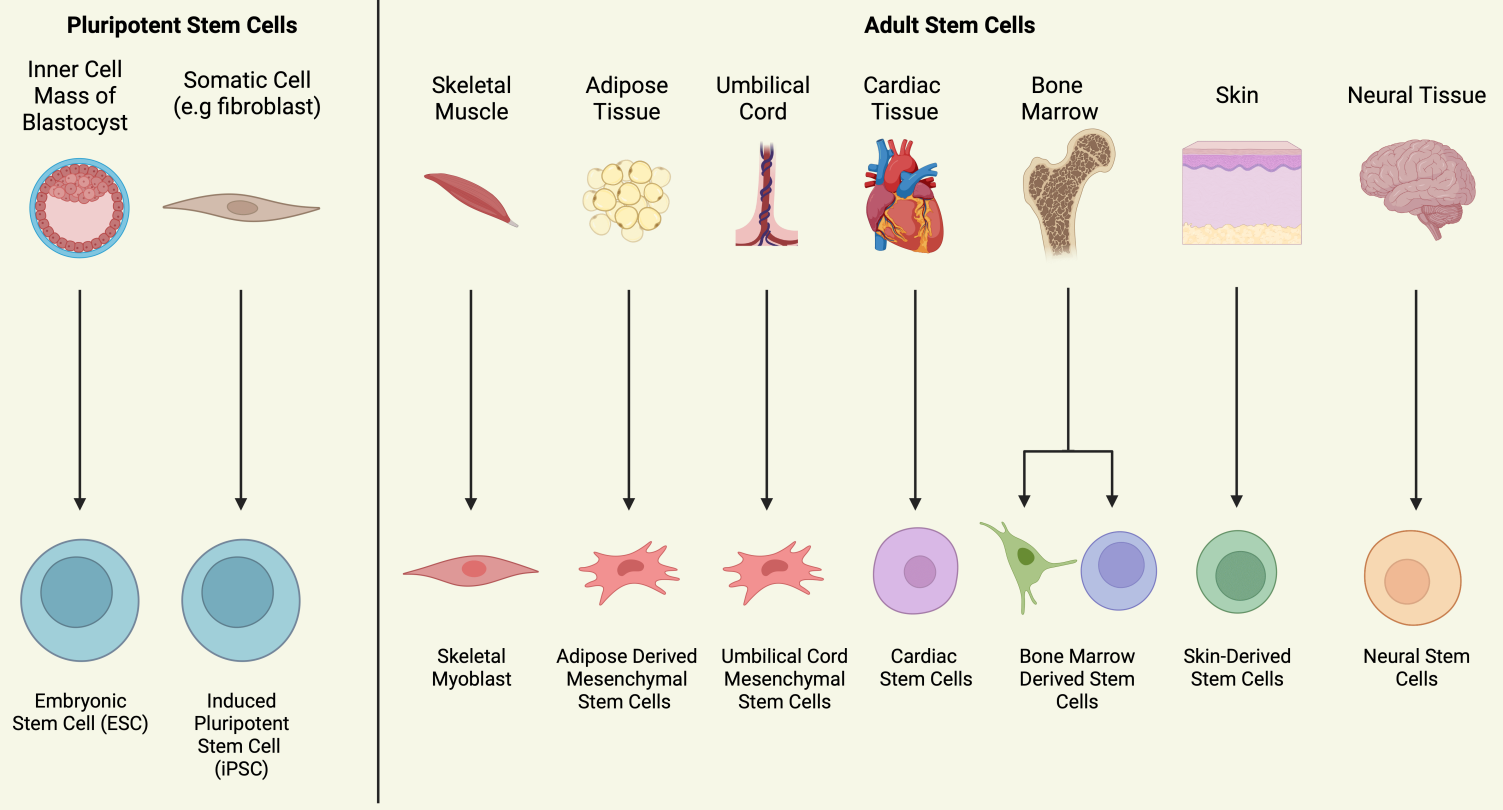
**Sources of Stem Cells Used in Cardiac Repair**. Figure summarising the most significant sources of stem cells used for cardiac repair. Pluripotent stem cells can be obtained from the inner cell mass or generated from somatic cells by introducing specific transcription factors. Adult stem cells exhibit multipotency and are derived from various adult tissues and organs.

Stem cells are undifferentiated and self-renewing, forming identical clones with the potential to differentiate into an array of specialised cell types, first discovered in 1961 [7]. Progenitor cells are the immediate descendants of stem cells, formed through asymmetric division, but instead give rise to tissue-specific progenitors [43]. Stem cells can be further categorised based on their degree of lineage commitment (Fig. 3). Embryonic stem cells (ESCs) exhibit pluripotency, while adult stem cells exhibit limited differentiation ability (multipotency) and less potent self-renewal [43]. As adult stem cells are derived from adult organs and tissues, they prove easier to obtain, allow for autologous transplantation and are not associated with the same degree of legal and ethical issues ESCs face. To be an ideal candidate for cardiac repair, stem cells should meet the following criteria; straightforward isolation, scalability into large quantities, the capacity to promote vascularisation, ability to reduce ischaemic imbalance and to differentiate into cardiac cell lineages (for integrating-based cell therapies), appropriate long-term electromechanical stability and integration within host myocardium and exertion of positive paracrine effects through the release of bioactive molecules including pro-angiogenic factors. This review will focus on those stem cells which have been extensively investigated for their use in IHD – these being embryonic stem cells (ESCs), induced pluripotent stem cells (iPSCs), skeletal myoblasts (SkMs), adipose derived mesenchymal stem cells (ASCs), umbilical cord mesenchymal stem cells (UCMSCs), endothelial progenitor cells (EPCs), cardiac progenitor cells (CPCs) and stem cells which reside in the bone marrow (often referred to collectively as bone marrow mononuclear cells (BMCs)), or separated into haemopoietic stem cells (HSCs) and bone marrow-derived mesenchymal stromal cells (BdMSCs). However, it should be noted that other adult stem cell populations exist including skin stem cells, epithelial stem cells and neural stem cells. Pre-clinical studies on the use of neural crest stem cells and amniotic epithelial cells for cardiac repair warrant further investigation, furthermore human dermal fibroblasts have been incorporated into synthetic scaffolds with iPSC dcrived cardiomyocytes in pre-clincial studies [44, 45, 46, 47].

With this as background, we will next review different types of stem cells that have been trialled for their efficacy to regenerate or repair the ischemic myocardium.

### 4.1 Embryonic Stem Cells

ESCs are pluripotent cells extracted from the inner cell mass of human blastocysts. They exhibit the capacity for indefinite symmetrical division; and for asymmetric division into progenitors of mesoderm, ectoderm and endoderm germ layers [48]. Owing to their pluripotency, ESCs are strong candidates for the repair of various adult tissue types including ischaemic myocardium. Several methods have been employed to induce cardiomyogenic differentiation from a pool of ESCs with the resulting cells termed embryonic stem cell-derived cardiomyocytes (ESC-CMs) [49]. ESC-CMs express early cardiac-specific transcription factors including homeobox protein Nkx2.5, GATA binding protein 4 (GATA-4), myocyte enhancer factor 2C (MEF2C) and T-box transcription factors Tbx-5 and Tbx-20 [50]. Furthermore, ESCs give rise to progenitor cells with temporal regulation of foetal liver kinase 1 (Flk1), Islet-1 (Isl-1) and Brachyury (a T-box transcription factor), demonstrating its capacity for differentiation into cardiomyocytes, endothelial cells and vascular smooth muscle cells *in vitro * [51, 52].

In a seminal study, Caspi *et al*. [53] transplanted undifferentiated ESCs into infarcted rat hearts, as it was thought the *in vivo* cardiac environment may be sufficient to induce cardiomyocyte (CM) differentiation. This proved unsuccessful, and teratomas consisting of cells from all three germ layers were observed, highlighting the importance of inducing CM differentiation and ensuring homogeneity of the cell population prior to transplantation, reducing the risk of tumorigenesis. In a follow-up study, ESC-CMs were grafted into the infarcted area 7–10 days following left anterior descending artery (LAD) ligation in a rat model. Improvements were noted in both scar remodelling and left ventricular function, with transplanted cells able to form gap junctions with host cells as assessed by the expression of the left ventricular gap junctional protein connexin-43. However, ESC-CMs exhibited an immature phenotype, which has been identified in subsequent studies from independent laboratories [54, 55]. ESC-CMs are observed as being smaller in size, having a slower action potential upstroke, a less extensive network of T-tubules and poor sarcomeric organisation among other issues [54, 55]. These differences to adult cardiomyocytes may impair the ability of ESC-CMs to effectively integrate within host myocardium, increasing the risk of arrythmia generation due to the difference in electrophysiology. Promising pre-clinical studies including the recent work of Liu *et al*. [56] have demonstrated that transplantation of ESC-CMs into infarcted myocardium in a non-human primate model improves left ventricular ejection fraction up to 3 months post-transplantation, however this was associated with an increased incidence of ventricular arrythmias.

To reduce the incidence of these events, current studies are aimed at enhancing the maturity of ESC-CMs *in vitro* before transplantation. Methods of improving maturation include inhibiting hypoxia-inducible factor 1-alpha (HIF-1α) and lactate dehydrogenase A (LDHA)—targeting metabolic maturity, along with bioreactor systems employing pulsatile flow, cyclic strain and extended culture time [57, 58].

In addition, transplantation of ESC-CMs into the recipient heart is allogenous, requiring lifelong immune system modulation [59]. Further, legal and ethical concerns exist as the traditional method of isolating ESCs for *in vitro* expansion results in the destruction of the human embryo [48]. While alternative, non-destructive approaches are being explored to isolate ESCs, these legal issues effectively rule out the use of ESCs as a viable treatment in many parts of the world.

Recently, the Transplantation of Human ESC Derived Progenitors in Severe Heart Failure (ESCORT) trial was completed (NCT02057900)—assessing the safety of ESC derived cardiac progenitor cells (CPCs) when engrafted into human patients [60]. Despite the knowledge that ESC-CMs exhibit an immature phenotype, the team behind this trial conducted several pre-clinical trials using these ESC derived CPCs, in which no arrythmia or teratoma formation was observed [61]. Six patients with severe ischaemic left ventricle (LV) dysfunction received ESC derived CPCs delivered as epicardial injection during coronary artery bypass graft (CABG) surgery and were followed up for a median of 18 months. All patients who were followed up had an uneventful recovery with no observed arrhythmia development. Importantly, a significant increase in systolic motion was observed in patients receiving ESC-CPCs. These results were encouraging despite a small sample size and warrant further investigation to confirm the efficacy of ESC derived CPCs in human populations. The on-going HECTOR study (NCT05068674) is a phase I trial evaluating the safety of administering varying doses of ESC-CMs to patients with LV dysfunction secondary to MI. This study aims to recruit 18 patients and will provide further insight into the safety profile of ESC-CM transplantation for cardiac repair.

In spite of these recent clinical trials, there are issues that need to be solved regarding the use of ESC derivatives for repair of the ischaemic heart. These include improving long-term engraftment rates, deciding the stage of differentiation for transplantation, increasing *in vitro* maturity of ESC-CMs and establishing the optimal dosage and safety profile. Further investigations are also required to accurately determine the nature and underlying mechanisms of the beneficial effects observed on ischaemic myocardium—whether this stems from remuscularisation of the heart or through paracrine mediated mechanisms.

### 4.2 Induced Pluripotent Stem Cells

In 2006, Takahashi *et al*. [62] generated pluripotent cells from adult fibroblasts and coined the term iPSCs, a discovery which would go on to win the Nobel Prize in Physiology or Medicine in 2012. As iPSCs are generated from somatic cells, they are not associated with the same ethical and legal issues as ESCs. Furthermore, patient-matched autologous cells can be created—thereby reducing the possibility of immune rejection. The most common method of iPSC production is the viral transduction of genes encoding the transcription factors octamer-binding protein 3/4 (Oct-3/4), sex-determining region Y box 2 (SOX2), Myc proto-oncogene protein (c-Myc) and Kruppel-like factor 4 (KLF4) [62]. Several studies have documented the ability to differentiate iPSCs into cardiomyocytes (iPSC-CMs), with the differentiation efficacy being comparable to that of ESCs, with ESC-CMs and iPSC-CMs sharing near identical transcriptional profiles [63, 64, 65]. In recent years, methods for the derivation and purification of cardiomyocytes from iPSCs have improved, with some studies reporting a purity of the differentiated cell pool for cardiomyocytes of around 95%, with 100 cardiomyocytes generated per input cell [66, 67].

Despite these advantages, differentiation of iPSCs poses some risks. Viral transduction carries with it the inherent risk of genome insertion at unwanted locations, with the potential to promote oncogenesis and disrupt cellular function [68]. As a result, non-viral vectors (e.g., plasmids) have been developed for iPSC production along with RNA and protein delivery. However, these alternate methods tend to be less efficient in iPSC production, as summarised in a review by Rao *et al*. [69]. While the ability for autologous transplantation using iPSCs is an advantage, this process can be costly and time consuming, with recent studies instead attempting to reduce the immunogenicity of allogenic iPSCs from healthy donors for clinical application [70]. Differentiation of iPSCs into CMs *in vitro* is achieved through similar protocols to those established for ESC differentiation, with the resulting CMs sharing an immature phenotype and therefore also carrying the risk of arrythmia and teratoma development [55]. This was highlighted in a recent pre-clinical trial conducted by Shiba* et al*. [71], in which iPSC-CMs were delivered by intra-myocardial injection into the infarcted hearts of non-human primates. Significant improvements in contractile function were reported, however with an increased incidence of ventricular tachycardia in the treatment group.

In a recently published first-in-human clinical trial in Japan, a 51-year-old male with severe ischaemic cardiomyopathy underwent transplantation of three allogenic iPSC-CM patches onto the ischaemic myocardium [72]. These patches were constructed of clinical grade iPSC-CMs which underwent screening for tumorigenesis and arrhythmogenesis risk. Following transplantation, no tumour development, arrythmias or effects related to immunosuppressive treatment were observed. Furthermore, the patient experienced increased quality of life, systolic motion, reduced LV global wall stress (attenuation of fibrosis) and an increase in the coronary flow reserve after 6 months and one year of follow up. The investigators hypothesized the observed benefit was primarily mediated through paracrine stimulation of angiogenesis. In support of this, a pre-clinical study conducted by Tachibana *et al*. [73] provided evidence that iPSC-CMs release high levels of interleukin-8 (IL-8), granulocyte colony stimulating factor (GCSF) and vascular endothelial growth factor (VEGF) promoting angiogenesis in the ischaemic heart. While the first-in-human clinical trial is promising, the extent of mechanical contribution of the engrafted iPSC-CMs to cardiac contractility and the percentage of cell retention necessitates further investigation. While no randomised clinical trials of iPSC-CM efficacy have been completed, one trial registered in China (NCT03763136) aims to assess the safety, feasibility, and efficacy of intramyocardial delivery of allogenic iPSC-CMs at the time of CABG surgery in patients with CHF. Another registered clinical trial (NCT03759405) will determine changes in quality of life and cardiac function following intravenous injection of iPSC-CMs in three patients with CHF. The recruitment has not started yet, with the trial expected to be completed in 2024. Although early results from case-studies are encouraging, further research needs to be carried out to standardize the protocol for developing mature cardiomyocytes from iPSCs that can electromechanically couple with endogenous cardiomyocytes for its clinical translation to be successful.

### 4.3 Skeletal Myoblasts

SkMs are multipotent stem cells located between the basal lamina and sarcolemma layers of mature skeletal muscle, becoming activated in response to muscle damage and degeneration [74]. SkMs were one of the earliest adult stem cell types explored as a candidate for cardiac repair due to their ability to form mature myofibers, resistance to ischaemia (owing to their skeletal muscle origin), relative ease of harvest and the potential for subsequent autologous transplantation [75, 76].

Pre-clinical murine studies including the pioneering work of Taylor* et al. * [77] demonstrated beneficial effects of SkM transplantation in improving cardiac contractility, preventing left ventricular remodelling, and decreasing diastolic pressures [76, 77, 78]. However, SkMs are committed to a myogenic lineage and therefore cannot form fully functioning cardiomyocytes, instead differentiating into muscle fibres called myotubes *in vivo * [79]. Cardiomyocytes behave as an electrical syncytium due to the presence of intercalated discs, more specifically gap junctions between neighbouring cells [80]. Myotubes derived from SkMs fail to form these gap junctions owing to a decreased expression of the intracellular adhesion molecules N-cadherin and connexin-43, therefore remaining electromechanically isolated from the host myocardium [79]. Thus, myotubes do not contract in synchrony with the surrounding myocardium, predisposing the transplant recipient to arrythmia development. Studies using skeletal myoblasts genetically modified to overexpress connexin-43 demonstrated improved myocardial integration and synchronous contractility stemming from an increase in the number of gap junctions formed with host cardiomyocytes [81, 82, 83]. However, subsequent studies have concluded this is insufficient in preventing the development of arrhythmias [84]. Furthermore, the Myoblast Autologous Grafting in Ischaemic Cardiomyopathy (MAGIC) phase II clinical trial showed no improvement in left ventricular ejection fraction (LVEF) in patients treated with skeletal myoblasts compared with the control group, with a higher number of arrhythmic events observed in the SkM-treated group [85]. Due to these major issues, skeletal myoblasts have not progressed as a candidate for cardiac repair.

### 4.4 Bone Marrow-Derived Stem Cells

BMCs are isolated from bone marrow throughout the body and can be further divided into HSCs and BdMSCs. HSCs exhibit myeloid and lymphoid differentiation, whereas BdMSCs differentiate into bone, cartilage and adipose tissue lineages *in vivo * [86, 87]. Unfortunately, many clinical trials using BMCs have not specified which sub-population was used, and definitions of these cells remain controversial. This section will focus on studies reportedly using HSCs, and more generally BMCs collectively. BdMSCs will be described in further detail in subsequent sections. One of the earliest studies involving BMC transplantation for repair of the ischaemic heart was conducted by Orlic* et al*. [88], who injected BMCs enriched for ${\mathrm{Lin}}^{-}$${\mathrm{c-kit}}^{\text{POS}}$ cells (used to distinguish HSCs) into the infarcted left ventricle of mice. The group reported direct transdifferentiation of BMCs into cardiomyocytes, endothelial cells and smooth muscle cells. The same group provided evidence that the administration of stem cell factor (SCF) and granulocyte-colony stimulating factor (G-CSF) induced mobilisation of BMCs to the infarct area, improving post-MI survival and supporting myocardial repair in a mice model [89]. While these findings were encouraging, landmark contradictory studies conducted by Balsam *et al*. [90] and Murry *et al*. [91] demonstrated that when injected into the myocardium of mice post-MI, HSCs did not improve survival *or* reduce infarct size. Furthermore, no cases of transdifferentiation into cardiac lineages were observed [91, 90]. The commonly accepted mechanism of repair is that BMCs exert their beneficial effects on ischaemic myocardium through the activation of endogenous progenitor cells, and the release of paracrine mediators including VEGF, basic-fibroblast growth factor (bFGF) and angiopoietin-1 (Ang-1) [92, 93]. Preclinical studies demonstrating the efficacy of BMCs for cardiac repair prompted quick clinical translation into randomised controlled trials in human populations [88, 89, 92, 93, 94].

The Reinfusion of Enriched Progenitor Cells and Infarct Remodelling in Acute Myocardial Infarction (REPAIR-AMI) study was a placebo-controlled phase III trial assessing the efficacy of BMCs delivered via intracoronary infusion following acute MI. This study demonstrated a significant improvement in left ventricular ejection fraction (LVEF) in the treatment group [95]. The PreSERVE-AMI study was a phase II study in which autologous purified ${\mathrm{CD34}}^{+}$ cells (a subset of BMCs) or a control were administered via intra-coronary infusion to STEMI patients [96]. After one year, while adjusting for the duration of ischaemia a dose-dependent improvement was observed in LVEF, infarct size and survival—suggesting purified ${\mathrm{CD34}}^{+}$ cells may have beneficial effects on the ischaemic heart. Although these two trials reached positive conclusions, several other trials have produced contradictory findings demonstrating little or no beneficial effect [97, 98, 99, 100]. BMCs have been extensively studied in human trials for cardiac regeneration over the last two decades, with around 100 randomised controlled trials (RCTs) produced. Meta-analyses of these RCTs has proven difficult due to the heterogeneity of methods used for cell production, administration, and measurements of cardiac performance [101, 102]. Despite this, BMCs remain an attractive candidate for cardiac repair. To draw robust conclusions, a greater number of well performed RCTs are required with consistent methodology and clearly defined cell populations.

### 4.5 Mesenchymal Stromal Cells

Mesenchymal stromal cells (MSCs) are multipotent with potential for differentiation into mesenchymal lineages (osteoblasts, chondrocytes, myocytes, adipocytes and fibroblasts), as defined by the International Society for Cell & Gene Therapy [103]. MSCs are attractive candidates for therapeutic cell transplantation due to their low immunogenicity and immunomodulatory capacity resulting from low major histocompatibility complex (MHC) II expression and the secretion of several anti-inflammatory cytokines. This increases the feasibility of allogenic MSC transplantation as an attractive option for large scale clinical implementation [104]. MSCs isolated from several sources have been investigated for their use in cardiac repair. The following sections will focus on bone-marrow derived mesenchymal stem cells along with MSCs derived from adipose tissue and the umbilical cord.

#### 4.5.1 Bone Marrow Derived Mesenchymal Stromal Cells

Bone marrow contains a population of BdMSCs with the capacity for differentiation into osteogenic, chondrogenic and adipogenic lineages [103]. Several studies have trialled the isolation and expansion of this cell population for administration into ischaemic or infarcted myocardium. One of the earliest *in vivo* studies investigating BdMSCs for cardiac repair was conducted by Toma *et al*. [105]*,* who injected lacZ-labelled human BdMSCs into the left ventricle of adult mice. Despite a high cellular attrition rate, the surviving BdMSCs began to resemble neighbouring cardiomyocytes and expressed proteins classically found within CMs including troponin T (cTnT), a-actinin and desmin. In a subsequent landmark clinical trial, Chen *et al*. [106] randomised sixty-nine participants who underwent PCI for acute MI to receive intra-coronary administration of autologous BdMSCs, or a saline control at 18 days post-PCI. After three months of follow-up - reduced perfusion defects, decreased left ventricular end-systolic and end-diastolic volumes, along with an increased LVEF were observed in the BdMSC treatment group.

Recently, Lee *et al*. [107] conducted a randomised, but open–label study assessing the safety and efficacy of autologous BdMSCs administered into the affected coronary artery at 1-month post-MI (n = 80). After a six-month follow up period, significant improvements were noted in the LVEF of participants who received BdMSCs. Additionally, no serious adverse effects were observed during the procedure or in the six months that followed.

Due to low MHC-II expression, transplantation of allogenic BdMSCs is also a possibility [104]. In a randomised, double blinded, placebo-controlled study, Hare *et al*. [108] provided evidence that administration of allogenic BdMSCs is not associated with an increased rate of adverse cardiac events, and furthermore improved left ventricular function and attenuated cardiac remodelling. Despite beneficial effects of BdMSC transplantation observed in these clinical trials, there is an evident lack of long-term clinical trials (>1 year) to determine whether these effects are sustained.

As BdMSCs and BMCs are both derived from bone marrow, interest lies in determining which of these cell populations is more efficacious for repair of the ischaemic heart. A meta-analysis of clinical trials conducted by Hosseinpour* et al*. [109] revealed that although both cell types significantly increase LVEF following MI, BdMSCs were more effective in improving cardiac contractility. Currently, a registered phase II, randomised, double-blinded placebo-controlled trial is aiming to assess functional improvement in ${\mathrm{VO}}_{\text{2MAX}}$ (maximum rate of oxygen consumption) after intramyocardial administration of autologous BdMSCs—the Administration of Mesenchymal Stem Cells in Patients with Ischaemic Cardiomyopathy (MESAMI2) study (NCT02462330). Results from this study will add to the growing pool of evidence surrounding the use of BdMSCs for IHD, and inform future directions.

#### 4.5.2 Adipose-Derived Stromal Cells

ASCs are located within deposits of adipose tissue, where they comprise around 5% of the cell population and are characterised by expression of the same surface markers as BdMSCs, while also being ${\mathrm{CD31}}^{-}$ and ${\mathrm{CD34}}^{+}$ [110]. Their efficacy has been extensively investigated in ischaemic conditions owing to their pro-angiogenic potential in pre-clinical models [111, 112, 113]. A heterogenous population of human ASCs were first isolated by Zuk *et al*. [114] in 2002 from lipoaspirate waste. The isolation of ASCs is minimally invasive with higher yields than the isolation of BdMSCs (5% vs 0.001%) [115]. This may allow for autologous transplantation without prior *ex vivo* expansion if sufficient cell numbers can be obtained. While ASCs exhibit the potential for differentiation into cardiac linages *in vitro *after supplementation with various factors, secretion of therapeutic paracrine factors with angiogenic, cardioprotective and anti-apoptotic effects including VEGF, hepatocyte growth factor (HGF), insulin-like growth factor 1 (IGF-1) and a variety of beneficial microRNA are thought to be the major contributors to ASCs induced improvements in cardiac performance [116, 117, 118, 119, 120, 121, 122, 123, 124, 125, 126].

Clinical trials investigating the efficacy of ASCs for repair of the ischaemic heart have recently begun. In 2014, the placebo-controlled double blinded Adipose-Derived Regenerative Cells in Patients with Ischaemic Cardiomyopathy (PRECISE) study tested the feasibility of autologous trans-endocardial ASC administration to patients with IHD [127]. Safety endpoints were followed for 36 months, with no significant differences found between the ASC and placebo groups, although both wall motion and viable LV mass significantly increased in the treated group. This study was followed by the Autologous Adipose-Derived Regenerative Cells for Refractory Chronic Myocardial Ischaemia with Left Ventricular Dysfunction (ATHENA) trial, which randomised 31 patients to receive intramyocardial injections of either autologous ASCs or placebo [128]. Results showed a trend towards improvement in ${\mathrm{VO}}_{\text{2MAX}}$ and quality of life, although no difference in LV function, structure or volume was identified between the groups. A potential reason for a lack of improvement in LV function may be that a higher dose of ADSCs is required. As results from both of these trials support the feasibility and safety of ASCs as a therapy for IHD, investigation of the efficacy of ASCs has progressed to a phase II trial: Stem Cell Therapy in IschEmic Non-treatable Cardiac Disease (SCIENCE), with results yet to be announced [129].

#### 4.5.3 Umbilical Cord Mesenchymal Stromal Cells

The umbilical cord is a rich source of UCMSCs with the capacity for differentiation into osteogenic, adipogenic and chondrogenic lineages [130, 131]. UCMSCs present an attractive candidate for repair of the ischaemic heart due to their high proliferative potential, with longer telomeres/less cellular aging than other MSCs cell types [132]. Similar to other mesenchymal stromal cells, UCMSCs display low immunogenicity resulting from a lack of human leukocyte antigen DR (HLA-DR), CD80 and CD86 and have strong immunomodulatory and anti-inflammatory properties [133].

Similar to other MSCs, USMSCs exhibit the capacity for *in vitro* differentiation into cardiac linaeges [134, 135, 136]. *In vivo*, the likely mechanism of cardiac repair exerted by UCMSCs is the release of paracrine mediators including transforming growth factor beta 3 (TGF-β3) and HGF protecting against apoptosis, cardiac remodelling and improving angiogenesis [137, 138].

The Intravenous Infusion of Umbilical Mesenchymal Stem Cells in Patients with Heart Failure (RIMECARD) trial was a phase I/II study investigating the safety and efficacy of allogenic UCMSCs delivered via intravenous infusion to heart failure patients with reduced ejection fraction (HFrEF) (n = 30) [138]. UCMSCs showed no difference in adverse event rate compared to placebo group, and further no alloantibodies were identified. Significant improvements were observed both in LVEF and quality of life at 12 months of follow up. Future randomized trials include the WANICHD trial (NCT04551456), which aims to recruit 300 participants to investigate UCMSCs efficacy as anti-inflammatory agents in coronary artery disease, and the hUC-MSC trial (NCT04939077) (n = 20) that aims to provide further evidence on the safety and effectiveness of UCMSCs in the treatment of heart failure.

All three MSC types (BdMSCs, ASCs and UCMSCs) discussed here share the benefits of low immunogenicity and immunomodulatory properties. BdMSCs and ASCs have an inherent advantage due to the potential for autologous transplantation, while UCMSCs have demonstrated promise owing to their shorter telomeres and comparatively higher proliferative potential. Furthermore, all MSC types are essentially devoid of ethical issues—furthering their potential for clinical application. In order to determine which MSC type is more efficacious for cardiac repair, clinical trials must be conducted comparing the cell types, and in combination with one another.

### 4.6 Endothelial Progenitor Cells

EPCs exhibit the capacity for both direct endothelial differentiation and maintenance of existing vasculature through paracrine mediated mechanisms [139, 140]. However, substantial debate exists in the literature regarding the classification and origin of EPC sub-populations. Early studies suggested that EPCs originate from the bone marrow, which has recently been challenged [141, 142]. Although the exact developmental origin of EPCs remains unknown, major sources include both peripheral blood and umbilical cord blood [143].

Two distinct EPC populations have been identified and termed ‘early’ and ‘late’ EPCs [140]. Early EPCs refer to cells of a haemopoietic origin, now termed myeloid angiogenic cells (MACs). These cells express CD45, CD14 and CD31 while being negative for CD146 and CD133 [144]. The major mechanism of endothelial repair from MACs is the release of paracrine mediators promoting angiogenesis including VEGF [145]. Late EPCs are now referred to as endothelial colony forming cells (ECFCs) and are considered the ‘true’ endothelial progenitors. ECFCs display an endothelial phenotype, and are identified by their expression of CD146, CD31 and CD105 while being negative for CD45 and CD14 [144]. It is thought that ECFCs primarily exert their beneficial effects on the vasculature by direct differentiation into endothelial cells, profoundly contributing to de novo blood vessel formation and angiogenesis [145, 146]. The heterogeneity of definitions and the lack of a clear unambiguous marker of EPCs has made it increasingly difficult to draw valid conclusions from trials.

In one of the only completed clinical trials investigating EPCs for repair of the ischaemic heart in humans, Zhu *et al*. [147] studied the safety and efficacy of EPCs pre-treated with thymosin beta-4 in patients with acute ST segment elevation MI. After six months of follow-up, patients treated with EPCs exhibited increased walking distance and significant improvement in cardiac function compared to the control group. Interestingly, despite these positive results, the use of EPC in the clinical setting for IHD has not advanced further. The primary issues holding back the clinical investigation of EPC therapy include the aforementioned lack of clear and consistent phenotypic classification, extended duration of *in vitro* expansion due to their low occurrence, and high immunogenicity of the cells [139, 144].

### 4.7 Cardiac Progenitor Cells

The longstanding dogma that adult mammalian heart was traditionally viewed as a post-mitotic organ with little capacity for self-renewal was challenged in the early 2000s, when a group of researchers identified a population of cells in the myocardium of rats exhibiting classical features of stem cells [148]. These cells were identified as expressing the tyrosine kinase receptor c-kit while being negative for common haemopoietic lineage markers such as CD34. Although a number of early studies demonstrated the efficacy of c-${\mathrm{kit}}^{+}$ cells to differentiate into cardiomyocytes, this was challenged by later studies due to the inability to reproduce earlier results, and several early papers have been retracted [149, 150, 151, 152, 153, 154, 155]. It has since been established that transplanted ${\mathrm{c-kit}}^{+}$ cells mediate their beneficial effects on ischaemic host myocardium through the release of paracrine factors, with one study proposing that c-${\mathrm{kit}}^{+}$ cells are a cardiac endothelial cell population rather than an endogenous CPC population [153, 156, 157, 158].

In parallel to research focusing on c-${\mathrm{kit}}^{+}$ cells, research has also progressed in determining the efficacy of cardiosphere derived cells (CDCs) for the treatment of ischemic heart disease. Cardiosphere-forming cells are isolated from the explant outgrowth through enzymatic digestion. CDCs are subsequently derived from cardiospheres [159]. They are characterised by their unanimous expression of the surface marker CD105 (part of the TGFβ receptor complex) and CD90, while being negative for the haemopoietic marker CD45 [160, 161]. CDCs exhibit the capacity for differentiation into endothelial cells, cardiomyocytes and smooth muscle cells *in vitro*, but are thought to exert their beneficial effects on ischaemic myocardium primarily through the release of paracrine mediators promoting angiogenesis, activating EPCs and encouraging cardiomyocyte proliferation while suppressing LV remodelling, apoptosis and inflammation [162, 163]. The phase I, randomised dose-escalation Cardiosphere-Derived Autologous Stem Cells to Reverse Ventricular Dysfunction (CADUCEUS) trial assessed the safety and efficacy of autologous intracoronary CDCs infusion to HFrEF patients 2–4 weeks post-MI. Patients treated with CDCs showed a significant reduction in infarct size along with both improved viable heart mass and regional contractility with no increase in adverse events relative to the control group. However, changes in end diastolic volume (EDV), end systolic volume (ESV) and LVEF did not differ between treatment and control groups after six months [164]. Due to the encouraging results of the CADACEUS trial, this was followed by a large multicentre randomized, double-blinded placebo-controlled phase I/II trial - Allogeneic Heart Stem Cells to Achieve Myocardial Regeneration (ALLSTAR) [165]. This study used allogenic CDCs as they are a more viable and cost-effective treatment option. After one year of follow-up no safety concerns were raised. Furthermore, patients treated with CDCs exhibited a decreased infarct size, increased viable myocardial mass and improved regional function of the ischaemic myocardium. These positive results warrant continued investigation into their efficacy of CDCs for the treatment of IHD.

In addition to c-${\mathrm{kit}}^{+}$ and CDCs, few other CPCs populations such as islet-1 and cardiac side population cells have exhibited their potential to repair the ischemic heart in preclinical models, although further studies are required before they can be considered in human populations [166, 167, 168].

## 5. Mechanisms of Stem Cell Induced Cardiac Repair

**Fig. 4.**
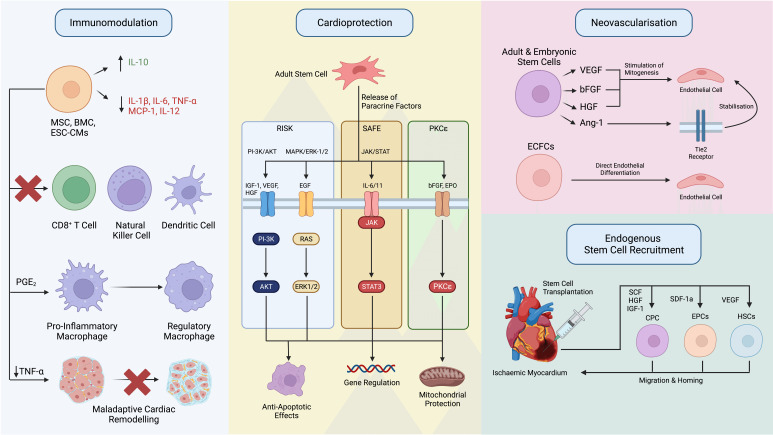
**Stem Cell Mechanisms for Cardiac Repair**. Summary of the proposed mechanisms for cardiac repair by transplanted stem cells. Stem cells secrete various factors with a paracrine effect on other cells promoting cardioprotection, neovascularisation, immunomodulation and endogenous stem cell activation, along with an autocrine feedback effect to enhance their survival. IL-10, interleukin 10; IL-1β, interleukin one beta; IL-6, interleukin 6; TNF-α, tumour necrosis factor alpha; MCP-1, monocyte chemoattractant protein 1; IL-12, interleukin 12; PGE2, prostaglandin E2; RISK, reperfusion injury salvage kinase; SAFE, surviving factor enhancement; PKCε, protein kinase C epsilon; PI3K, phosphoinositide 3-kinase; MAPK, mitogen-activated protein kinase; ERK, extracellular signal-related kinase; JAK, janus tyrosine kinase; STAT, signal transducer and activator of transcription; IGF-1, insulin-like growth factor-1; VEGF, vascular endothelial growth factor; HGF, hepatocyte growth factor; EGF, epidermal growth factor; IL-11, interleukin-11; bFGF; basic fibroblast growth factor; EPO, erythropoietin; RAS, rat sarcoma virus protein; STAT3, signal transducer and activator of transcription 3; Ang-1, angiopoietin-1; Tie2, tyrosine kinase with immunoglobulin-like loops and epidermal growth factor homology domains-2; SCF, stem cell factor; CPC, cardiac progenitor cell; SDF-1α, stromal cell-derived factor 1-alpha; EPC, endothelial progenitor cell; HSC, haemopoietic stem cell; ECFC, endothelial colony-forming cell.

The preclinical studies and clinical trials discussed in this review have provided evidence demonstrating the efficacy of stem cells for cardiac repair. However, the nature of this repair has been a focal point in recent years—with a shift from a theory of cell differentiation and remuscularisation towards paracrine mediated repair. This stems from an inconsistent ability to demonstrate differentiation of transplanted stem cells into cardiac linages *in vivo*, while nonetheless observing beneficial effects on cardiac contractility and coronary artery reserves [153, 169]. Furthermore, long-term improvements in cardiac performance have been observed at time points where very few transplanted cells remain, and where scar tissue separates the resident and transplanted cells [152]. To date, comparatively little is known about the paracrine effects of pluripotent stem cells, with most published research focusing on adult stem cells (MSCs, EPCs, CPCs, BMCs) – of which MSC sare the most extensively studied. The following sections will review our current understanding of the released paracrine factors and their beneficial effects on ischaemic myocardium (Fig. 4).

### 5.1 Immunomodulation

While the initial immune response to ischaemic injury is physiologically essential, a sustained inflammatory response is a direct contributor to adverse cardiac remodelling and progression to CHF [170, 171]. Both adult stem cells including MSCs, as well as ESC-CMs, secrete a plethora of anti-inflammatory cytokines which act to limit deleterious, sustained endogenous inflammation of the myocardium (Fig. 4). In particular, administration of these cells downregulates the expression of the pro-inflammatory cytokines tumour necrosis factor alpha (TNF-α), interleukin 1 beta (IL-1β), interleukin 6 (IL-6) and monocyte chemoattractant protein 1 (MCP-1) which play a role in LV remodelling [172, 173, 174]. In contrast, EPCs are known to release pro-inflammatory cytokines including MCP-1 and IL-8 [175]. TNF-α acts through tumour necrosis factor (TNF) receptors present on all cell types within the myocardium. Once activated, this induces matrix metalloproteinases (MMP)—proteins which break down the extracellular matrix (ECM) and are implicated in maladaptive cardiac remodelling [176]. MSC administration has further been shown to influence various immune cell populations in the heart after ischaemic injury. MSCs inhibit the cytotoxic activity of ${\mathrm{CD8}}^{+}$ T cells and natural killer (NK) cells, prevent dendritic cell maturation and have the capacity to either enhance or inhibit plasma cell immunoglobulin G (IgG) production depending on the signal intensity [177, 178]. In addition, soluble factors produced by MSCs including prostaglandin E2 (PGE2) have been demonstrated to switch the phenotype of pro-inflammatory macrophages to regulatory, anti-inflammatory macrophages with the capacity to promote angiogenesis [179]. Furthermore, MSCs show decreased production of the pro-inflammatory IL-12, and an increased production of anti-inflammatory cytokines including interleukin 10 (IL-10) [180, 181]. Collectively, this immune system modulation is an important part of how stem cells mediate their beneficial effects within ischaemic myocardium following transplantation.

### 5.2 Cardioprotection

Cardiomyocyte apoptosis is a significant contributor to ischaemic injury and maladaptive cardiac remodelling [182]. Thus, protecting cardiomyocytes from apoptosis may attenuate ischaemic injury while promoting their proliferation. Cultured adult stem cells including BMCs, MSCs and c-${\mathrm{kit}}^{+}$ CPCs have been shown to secrete factors including interleukin-11 (IL-11), VEGF, erythropoietin (EPO), fibroblast growth factor-2 (FGF-2), IGF-1, HGF and epidermal growth factor (EGF). These molecules activate several pro-survival pathways, improving cardiomyocyte survival in the hostile environment of ischaemic myocardium (Fig. 4) [156, 183, 184, 185].

Most of the above secreted factors function through the activation of pro-survival kinases Akt and ERK1/2, the downstream signalling cascade of phosphatidylinositol 3-kinase (PI3K) and mitogen-activated protein kinase (MAPK) signalling respectively (PI3K/AKT, MAPK/ERK-1/2), collectively known as the reperfusion injury salvage kinase (RISK) pathway [186, 187, 188, 189]. Acute activation of this pathway mediates cardioprotection, however chronic activation can result in cardiac hypertrophy [190]. A study conducted by Noiseux *et al*. [169] provided evidence that in rats, activation of the Akt signalling pathway by BdMSCs conferred improved efficacy in enhancing cardiomyocyte survival and preventing apoptosis following myocardial infarction.

Other pathways involved in cardioprotection by stem cells include the surviving factor enhancement (SAFE) pathway and the protein kinase c epsilon (PKCε) pathway. These pathways are activated by IL-6 and IL-11, along with bFGF and EPO secreted from transplanted stem cells. The SAFE pathway leads to activation of signal transducer and activator of transcription 3 (STAT3) through Janus-kinase (JAK) signalling [191, 192, 193]. STAT3 contributes to cardioprotection by inhibiting the opening of the mitochondrial permeability transition pore and thus apoptosis [194]. Among other targets, PKCε is known to activate aldehyde dehydrogenase 2 (ALDH2)—detoxifying reactive aldehydes, and further to interact with cytochrome-c oxidase, decreasing intracellular ROS, protecting against apoptosis [195].

### 5.3 Neovascularisation

Due to the imbalance between myocardial oxygen demand and supply in IHD, reperfusion of the ischaemic area is essential for improving clinical prognosis and halting the progression of IHD. Neovascularisation consists of angiogenesis (growth of endothelial sprouts from existing vessels), vasculogenesis (differentiation of angioblasts into endothelial cells) and arteriogenesis (smooth muscle migration, growth and remodelling) [196]. Secreted paracrine factors promoting neovascularization from BMCs, MSCs, c-${\mathrm{kit}}^{+}$ CPCs, human iPSC-CMs and EPCs include VEGF, bFGF, Ang-1 and HGF among others (Fig. 4) [93, 184, 197, 198, 199]. Both VEGF and bFGF have direct mitogenic effects on endothelial cells, promoting angiogenesis [200]. Ang-1 mediates its beneficial effects on the vasculature through Tie2 receptors where it is primarily involved in endothelial stabilisation [201]. In a similar fashion to bFGF, HGF has also been demonstrated to work synergistically with VEGF to promote endothelial cell survival, proliferation and tubulogenesis [202]. The importance of VEGF for mediating the beneficial effects of MSCs after transplantation into ischaemic myocardium was highlighted by a study where the VEGF gene was ablated. Following ablation, MSCs exhibited a significantly impaired ability to promote functional recovery in the ischaemic heart [203]. Along with paracrine potentiation of angiogenesis, ECFCs (a subset of EPCs) participate in de novo vasculogenesis by direct differentiation into endothelial cells [146].

### 5.4 Recruitment and Activation of Endogenous Stem Cells

Recent studies have demonstrated the ability of transplanted exogenous stem cells to activate resident and circulating stem cells, enabling endogenous cardiac regeneration (Fig. 4) [204]. While the specific paracrine factors responsible are yet to be identified, Urbanek *et al*. [205] showed that c-Met/HGF and IGF-1 receptors expressed by CPCs were able to activate resident CPCs, forming *de novo* myocardium in a murine model. In another study from an independent laboratory, administration of HGF and IGF-1 to CPCs isolated from a porcine model promoted CPC proliferation, migration and the activation of downstream signalling pathways including phosphorylation of Akt. Interestingly, HGF and IGF-1 seem to act synergistically, as the observed effects were far greater in combination than either alone [206]. When applied to CPCs obtained from the neonatal rat heart, MSC conditioned medium was able to improve CPC proliferation and inhibit apoptosis [204]. In addition, MSCs also express bone morphogenetic proteins (BMPs), Wnt pathway modulators and FGF, all of which are involved in regulating CPC differentiation and commitment, suggesting these molecules may contribute to the regeneration of the myocardium through the activation of endogenous stem cells [207]. Along with activation of CPCs, circulating stem cells including MSCs, BMCs and HSCs home to ischaemic myocardium following insult from the bone marrow and circulation [208, 209]. Further, studies have suggested the ability of transplanted MSCs to recruit circulating EPCs, c-${\mathrm{kit}}^{+}$ stem cells and ${\mathrm{CD34}}^{+}$ stem cells through the release of chemotactic homing factors stromal cell-derived factor 1 alpha (SDF-1α), SCF and VEGF respectively [210, 211, 212].

### 5.5 Activation of Transcription Factors

Transplantation of stem cells into ischaemic myocardium exposes them to a hypoxic environment, triggering activation of transcription factors which have pro survival and proliferative potential [213, 214]. A key transcriptional factor overexpressed in MSCs among others is hypoxia-inducible factor-1α (HIF-1α), playing a vital role in the cellular adaptation to ischaemia [213, 215]. Activation of HIF-1α leads to the downstream secretion of VEGF, platelet-derived growth factor (PDGF) and EPO which promote cell proliferation and angiogenesis, reducing apoptosis [216]. Interestingly, HIF-1α can also be stimulated by various hypoxia response pathways, including the PI-3K/AKT pathway [217].

## 6. Conclusions & Challenges

IHD and its various complications remain the leading cause of mortality worldwide. Despite advances in the discovery of novel therapeutics for IHD, no widespread clinical translation has occurred of a treatment able to regenerate ischaemic myocardium, thereby restoring cardiac function. It has become increasingly clear that cell-based therapies primarily exert their beneficial effects within ischaemic myocardium through the release of paracrine mediators, rather than remuscularisation of the heart. As outlined in this review, a number of cell-based therapies have demonstrated great promise in early clinical studies, with others including SkMs no longer investigated due to their adverse effects. To reach clinical translation, there is an immediate need to undertake clinical trials with larger sample sizes, a longer duration of follow up and clear, standardised phenotypic classification of cells.
